# Mitochondrial DNA Footprints from Western Eurasia in Modern Mongolia

**DOI:** 10.3389/fgene.2021.819337

**Published:** 2022-01-06

**Authors:** Irene Cardinali, Martin Bodner, Marco Rosario Capodiferro, Christina Amory, Nicola Rambaldi Migliore, Edgar J. Gomez, Erdene Myagmar, Tumen Dashzeveg, Francesco Carano, Scott R. Woodward, Walther Parson, Ugo A. Perego, Hovirag Lancioni, Alessandro Achilli

**Affiliations:** ^1^ Department of Chemistry, Biology and Biotechnology, University of Perugia, Perugia, Italy; ^2^ Institute of Legal Medicine, Medical University of Innsbruck, Innsbruck, Austria; ^3^ Department of Biology and Biotechnology “L. Spallanzani”, University of Pavia, Pavia, Italy; ^4^ Sorenson Molecular Genealogy Foundation, Salt Lake City, UT, United States; ^5^ FamilySearch Int., Salt Lake City, UT, United States; ^6^ Department of Anthropology and Archaeology, National University of Mongolia, Ulaanbaatar, Mongolia; ^7^ Department of Medical and Surgical Sciences, University of Bologna, Bologna, Italy; ^8^ Forensic Science Program, The Pennsylvania State University, State College, PA, United States; ^9^ Department of Math and Science, Southeastern Community College, Burlington, IA, United States

**Keywords:** Eurasian Steppe, Inner Asia, Mongolia genetic history, modern mitogenomes, mitochondrial DNA phylogeny, mtDNA haplogroups

## Abstract

Mongolia is located in a strategic position at the eastern edge of the Eurasian Steppe. Nomadic populations moved across this wide area for millennia before developing more sedentary communities, extended empires, and complex trading networks, which connected western Eurasia and eastern Asia until the late Medieval period. We provided a fine-grained portrait of the mitochondrial DNA (mtDNA) variation observed in present-day Mongolians and capable of revealing gene flows and other demographic processes that took place in Inner Asia, as well as in western Eurasia. The analyses of a novel dataset (N = 2,420) of mtDNAs highlighted a clear matrilineal differentiation within the country due to a mixture of haplotypes with eastern Asian (EAs) and western Eurasian (WEu) origins, which were differentially lost and preserved. In a wider genetic context, the prevalent EAs contribution, larger in eastern and central Mongolian regions, revealed continuous connections with neighboring Asian populations until recent times, as attested by the geographically restricted haplotype-sharing likely facilitated by the Genghis Khan’s so-called *Pax Mongolica*. The genetic history beyond the WEu haplogroups, notably detectable on both sides of Mongolia, was more difficult to explain. For this reason, we moved to the analysis of entire mitogenomes (N = 147). Although it was not completely possible to identify specific lineages that evolved *in situ*, two major changes in the effective (female) population size were reconstructed. The more recent one, which began during the late Pleistocene glacial period and became steeper in the early Holocene, was probably the outcome of demographic events connected to western Eurasia. The Neolithic growth could be easily explained by the diffusion of dairy pastoralism, as already proposed, while the late glacial increase indicates, for the first time, a genetic connection with western Eurasian refuges, as supported by the unusual high frequency and internal sub-structure in Mongolia of haplogroup H1, a well-known post-glacial marker in Europe. Bronze Age events, without a significant demographic impact, might explain the age of some mtDNA haplogroups. Finally, a diachronic comparison with available ancient mtDNAs made it possible to link six mitochondrial lineages of present-day Mongolians to the timeframe and geographic path of the Silk Route.

## Introduction

The Eurasian Steppe stretches from Europe to Inner Asia and represents an important crossroad in human history, characterized by migrations and admixtures of culturally and genetically distinct populations (Palstra et al., 2015). Mongolia covers most of the Eastern Steppes. Nowadays, it is a presidential republic divided into 21 provinces (*aimags*) and one provincial municipality (Ulaanbaatar); most of the population (71%) lives in urban centers, while the remaining 29%, often tied to nomadic lifestyles, lives in rural areas.

Archaeological evidence (Kovalev and Erdenebaatar, 2009; Wilkin et al., 2021) and genetic studies provided the first information on the complex Mongolian past (Cavalli-Sforza et al., 1994; Comas et al., 1998; Yao et al., 2004; Yang et al., 2008; Yunusbayev et al., 2015; Pugach et al., 2016; Bai et al., 2018). As in another population context (Achilli et al., 2018), archaeogenomics unveiled further details on the emerging scenario of admixture between Eastern and Western Eurasians for the origin of the Central Asian populations, with distinct west-east genetic gradients between different western and eastern Eurasian groups (Jeong et al., 2019; Narasimhan et al., 2019; Ning et al., 2021). In particular, recent analyses of ancient genomes spanning from 6000 before the common era (BCE) to present days revealed at least four ancestral sources that arose in Mongolia through the Neolithic. Two were identified in pre-Bronze Age individuals from northeastern and northern Mongolia and are associated to hunter-gatherer populations from northeast Asia and northern Eurasia, respectively; the third was connected with the Afanasievo culture, an eastward extension of the Yamnaya culture from the Pontic-Caspian steppe (ca. 3300–2200 BCE), which probably introduced dairying practices to the region (ca. 3000 BCE) and was later followed or replaced by the Chemurchek culture (2750–1900 BCE). A genetic mixture of Yamnaya pastoralists and European farmers, the fourth source, appeared ca. 1400 BCE (Jeong et al., 2020; Wang C. C. et al., 2021). During the Middle and Late Bronze Age (ca. 1900–900 BCE), ruminant dairying characterized by intensive nomadic herding without farming was widespread, leading to the development of large-scale polities since the late first millennium BCE. The Xiongnu was the first of different historically documented dynasties and empires, founded by pastoralists in the Early and Late Medieval periods such as Xianbei, Türk, Uyghur, Khitan, and Mongol. During those centuries Mongolia and the Eurasian Steppe represented an important crossroad through the notorious *Silk Road* (founded by the Chinese Han dynasty in 130 BCE) that played a major role in the economic, demographic and cultural processes shaping the history of several Eurasian populations (Comas et al., 1998). The Mongol empire arose in the late 12th century CE when the chieftain Temüjin took the title of *Genghis Khan* (*“Universal Ruler”*). At its peak (1206–1368 CE), the empire stretched from present-day Poland in the west to Korea in the east, and from Siberia in the north to the Gulf of Oman and Vietnam in the south, covering approximately 22% of Earth’s total land and with a population of over 100 million people. At the beginning, Genghis Khan used to destroy most infrastructures along the Silk Route, but eventually he decided to adopt a politics of supporting and facilitating commercial and cultural exchanges between regions under his dominion (Liu, 2010). The empire allowed the establishment of the *Pax Mongolica* (1280–1360 CE), indicating a pacific and flourishing period characterized by commercial, cultural, religious, and scientific exchanges between western and eastern populations, including trades between nomadic groups and urban centers (Köstenbauer, 2017). The historical stability under Genghis Khan’s rule is also supported when comparing genetic profiles of ancient Mongols with contemporary Mongolians (Zerjal et al., 2003; Bai et al., 2018).

Mitochondrial DNA (mtDNA) provided several pieces of information. Evidence deriving from the mtDNA haplogroups shared between Afanasievo and Yamnaya people supports an eastward migration from the Pontic-Caspian steppes (Allentoft et al., 2015; Narasimhan et al., 2019). The presence of a U5a1 mitochondrial haplotype in an Eneolithic grave, dated at ca. 3000 BCE and associated with the Afanasievo archaeological culture in the Khangai Mountains, attested the presence of people with “western” origin in the east of the Altai Mountains before the Bronze Age (Rogers et al., 2020), in contrast to what was previously proposed (Ricaut et al., 2004a; Ricaut et al., 2004b; Lalueza-Fox et al., 2004; Chikisheva et al., 2007; Keyser et al., 2009; González-Ruiz et al., 2012; Wang C. C. et al., 2021). To further investigate the impact and legacy of mitochondrial lineages with eastern and western origins on the gene pool of modern Mongolian populations, we analyzed the mtDNA profiles of 2,420 individuals with a last known terminal maternal ancestor (TMA) from one of the 20 different Mongolian provinces.

## Materials and Methods

### Sample Collection and DNA Extraction

A total of 2,420 biological samples belonging to unrelated subjects with a Mongolian TMA were collected in different areas of Mongolia, using 10 ml of commercially available mint-flavored mouthwash. Pedigree charts and informed consents were obtained from all participants. The samples were collected in 20 (out of 21) Mongolian provinces: Arkhangai (*n* = 4), Bayankhongor (*n* = 2), Bayan-Ölgii (*n* = 216), Bulgan (*n* = 5), Darkhan-Uul (*n* = 1), Dornod (*n* = 370), Dornogovi (*n* = 26), Dundgovi (*n* = 1), Govi-Altai (*n* = 8), Khentii (*n* = 132), Khovd (*n* = 429), Khövsgöl (*n* = 307), Ömnögovi (*n* = 2), Övörkhangai (*n* = 8), Selenge (*n* = 4), Sükhbaatar (n = 246), Töv (*n* = 10), Ulaanbaatar (*n* = 2), Uvs (*n* = 132), Zavkhan (*n* = 167); the remaining samples (*n* = 348) belonged to individuals who did not provide province information and therefore were associated to an “unspecified” group. Provinces with less than 30 individuals were grouped into three geographic macro-areas by considering their geographic position, biome, and orography: “Gobi Desert” (Bayankhongor, Dornogovi, Dundgovi, Govi-Altai and Ömnögovi), “Khangai Mountains” (Arkhangai and Övörkhangai), “Near Ulaanbaatar” (Bulgan, Darkhan-Uul, Selenge, Töv and Ulaanbaatar) (Supplementary Table S1).

### Mitochondrial DNA Control Region Analysis

DNA extraction and mtDNA control-region sequencing were performed as in Perego et al. (2012).

All resulting sequences have been deposited in GenBank under accession numbers OL632312-OL634731 and are also available in the EMPOP mtDNA population database (https://empop.online/) under accession number EMP00853. A total of 2,133 haplotypes encompassed the entire mitochondrial control region (CR, ∼1122 bps from np 16024 to np 576), while 2,335 haplotypes encompassed at least the HVS1 segment (nps 16024–16365). The sequences were aligned to the revised Cambridge Reference Sequence (rCRS; NC_012920.1) (Andrews et al., 1999) using Sequencher 5.10 (Gene Codes Corporation), in order to visualize electropherograms and identify and register any mutational differences. All samples were classified into haplogroups according to their respective mutational motifs by referring to PhyloTree build 17 (van Oven and Kayser, 2009). Considering the large number of maternal lineages identified in our study we grouped each of them into 18 macro-haplogroups (Supplementary Table S2).

Several mtDNA sequence variation parameters were estimated by using DnaSP 5.1 software (Librado and Rozas, 2009). Nucleotide diversity (π or Pi) and haplotype diversity (Hd) were plotted with Tableau 2021tbl 2021.3 onto Mongolian geographic map. In order to graphically display and summarize the mitogenetic relationships among the analyzed individuals, Principal Component Analyses (PCA) were performed using prcomp () from the stats R package (R Core Team, 2021) or the Excel software implemented by XLSTAT. Macro-haplogroup frequencies were used as input data. In intra-Mongolia analyses, the Khangai Mountains and the cosmopolitan “Near Ulaanbaatar” macro-area were excluded due to the low number (<30) of individuals in each of them.

The Mongolian maternal gene pool was further compared with a Eurasian dataset encompassing 546 bps of the control region (nps 16024–16569) obtained from 30,400 individuals from 69 countries/geographic areas (Supplementary Table S3). Four population groups outside Mongolia with less than 30 individuals were excluded from these analyses. The final dataset (for a total of 32,486 sequences including 2,133 Mongolian CR sequences from this study) was aligned with MEGAX (Kumar et al., 2018). The genetic distance between groups was also calculated with MEGAX using the p-distance (proportion of nucleotides at which two sequences being compared are different). The obtained distance matrix was used to construct a multidimensional scaling (MDS) using the R function cmdscale () (R Core Team, 2021). The heteroplasmic bases were converted to Ns with DNAsp 6 (Rozas et al., 2017), before calculating the haplotype sharing with Arlequin (Excoffier and Lischer, 2010). The ratio of haplotype sharing was calculated for each population pair by dividing the number of shared haplotypes by the total haplotypes in each paired group. The pairwise haplotype sharing ratio was also calculated separately for haplotypes belonging to eastern Asia (EAs) and western Eurasian (WEu) haplogroups.

### Complete Mitogenome Analysis

The entire mitogenome sequences of 147 individuals, representative of different macro-haplogroups, were obtained (Supplementary Table S4) by using the Ion Torrent Personal Genome Machine (PGM) and following manufacturers’ protocols. The entire mtDNA molecule was amplified with the HID-Ion Ampliseq Mitochondrial Library Preparation; then the template-positive Ion PGM Hi-Q Ion Sphere Particles were prepared with Ion OneTouch 200 Template Kit v2 and sequenced with the Ion PGM Hi-Q Sequencing Kit chemistry on an Ion 318 v2 chip using a multiplexing approach (Parson et al., 2013; Strobl et al., 2018).

The IGV (Integrative Genomics Viewer) software (Thorvaldsdóttir et al., 2013) was used to visualize the BAM files (aligned to the rCRS and produced by the sequencing machine aligning software) and to verify or to search for specific mutational differences throughout the entire mitochondrial genome. Ambiguous positions were analyzed by Sanger-type sequencing until clarity was reached. The quality of mitogenome sequences was checked through SAM2 on EMPOP (Parson and Dür, 2007; Huber et al., 2018), and all samples were classified into haplogroups according to PhyloTree build 17 (http://www.phylotree.org/) (van Oven and Kayser, 2009) using both Haplogrep 2.0 (Weissensteiner et al., 2016) and SAM2 on EMPOP. A few discrepancies using the two approaches were expected, as explained in Dür et al. (2021). However, these differences do not affect the outcomes of the present work. All control-region haplotypes were confirmed by the mitogenome sequences.

All complete mitogenomes (N = 147) are available in GenBank under accession numbers OL619795-OL619941 and in the EMPOP mtDNA population database (https://empop.online/) under accession number EMP00853.

The evolutionary relationships among our modern haplotypes were visualized through the construction of a most parsimonious (MP) tree, built with an updated version of mtPhyl v.5.003 and checked with MEGAX software. One published L3 sequence (accession number DQ341081) was used as outgroup to reconstruct time estimates and demographic trends in BEAST v2.6.6 (Bouckaert et al., 2019), as previously reported (Modi et al., 2020; Capodiferro et al., 2021). 95% of High Posterior Densities (HPD) were plotted for haplogroups younger than 20 thousand years ago (kya). A total of 693 published ancient mitogenomes from different Eurasian regions (including 25 excavated in Mongolia) were also analyzed taking into account haplogroup classification, age and location of the remains (Supplementary Table S5).

## Results

### The Mitochondrial DNA Variation Within Mongolia

**FIGURE 1:**
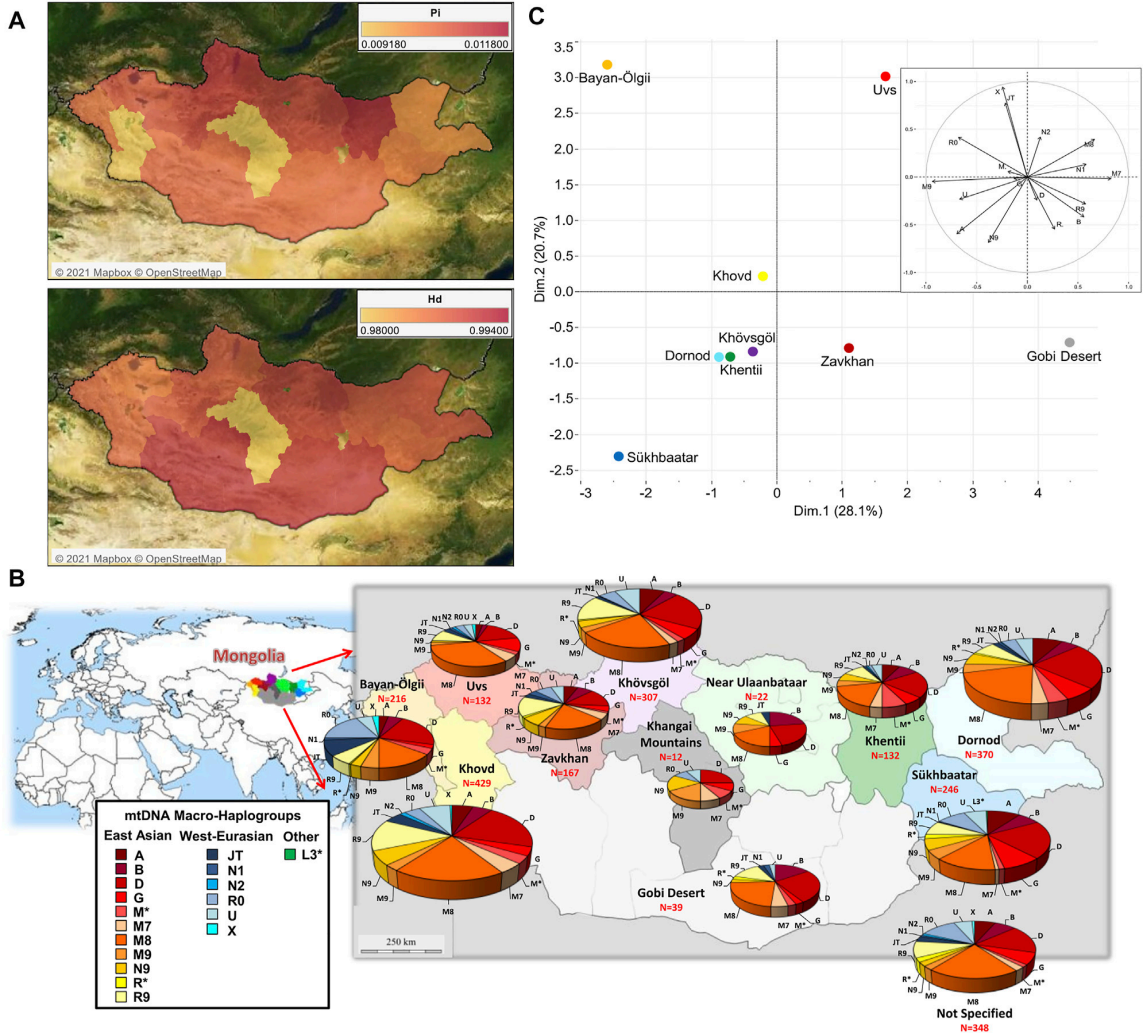
The mtDNA variation within Mongolia based on 2,420 modern control-region sequences. **(A)** Map of genetic variability in each macro-area expressed as nucleotide diversity (Pi) and haplotype diversity (Hd); this analysis was restricted to 2,335 complete HVS1 sequences. **(B)** Pie charts of the distribution of mtDNA macro-haplogroups. **(C)** PCA plot representing the genetic landscape of Mongolia based on macro-haplogroup frequencies; the following groups were excluded: “Khangai Mountains”, “Near Ulaanbaatar” and “Not Specified”.

The concomitant analysis of 2,335 HVS1 sequences from modern Mongolians identified 160 polymorphic sites (excluding gaps and ambiguous sites) with a Nei’s nucleotide diversity (π or Pi) of 0.00809, and a very high haplotype diversity (Hd = 0.986). This genetic diversity is heterogeneously distributed within Mongolia (Figure 1A). The “Near Ulaanbaatar” macro-area is characterized by high diversity values, probably due to very recent migrations towards regions around the capital. On the other hand, isolation could better explain the very low diversity in the Khangai Mountains, even if a sample bias due to the low number of individuals (N = 12) cannot be excluded. Interestingly, the Khovd region shows an average number of different haplotypes but a very low nucleotide diversity, which might indicate that people of this western area stretched between the Altai Mountains and the Gobi Desert brought different haplotypes in this area only recently, and their mitogenomes did not yet differentiate from each other.

The overall 2,420 Mongolian mtDNAs were classified into different 413 lineages and sub-lineages, ultimately grouped into 18 macro-haplogroups (A, B, D, G, JT, L3*, M*, M7, M8, M9, N1, N2, N9, R*, R0, R9, U, and X; Supplementary Table S2). The macro-haplogroup distribution across the country clearly shows a differential contribution of haplogroups with two distinct geographic origins (Figure 1B; Supplementary Table S1). As expected, most mtDNAs (1,987 out of 2,420: 82.1%) belong to eastern Asia (EAs) haplogroups with a notable incidence of C (19.6%) and D4 (19.8%) and higher frequencies in the eastern part of Mongolia. In the west, the presence of western Eurasian (WEu) haplogroups is significant (21.7%) and tends to decrease eastwards, but with the lowest occurrences in central regions. The most represented WEu haplogroup is H (6.5%), thus confirming the results obtained in previous studies on Inner Asia (Comas et al., 1998; Wells et al., 2001; Quintana-Murci et al., 2004; Derenko et al., 2014; Lan et al., 2019; Chen et al., 2020; Wang W. et al., 2021; Keyser et al., 2021). A geographic differentiation is clear in the PCA that represents the mtDNA genetic landscape of Mongolia (Figure 1C). The PC2 separates the westernmost regions, Bayan-Ölgii, Khovd and Uvs, due to the high contribution of the typical WEu lineages JT, N2, R0 and X. The northern regions (Dornod, Khentii and Khövsgöl) cluster together in the middle of the plot, while PC1 pushes the southern regions of Gobi Desert and Sükhbaatar apart from each other due to the differential distribution of typical EAs lineages.

### The Mitochondrial DNA Variation of Mongolia in the Eurasian Context

**FIGURE 2:**
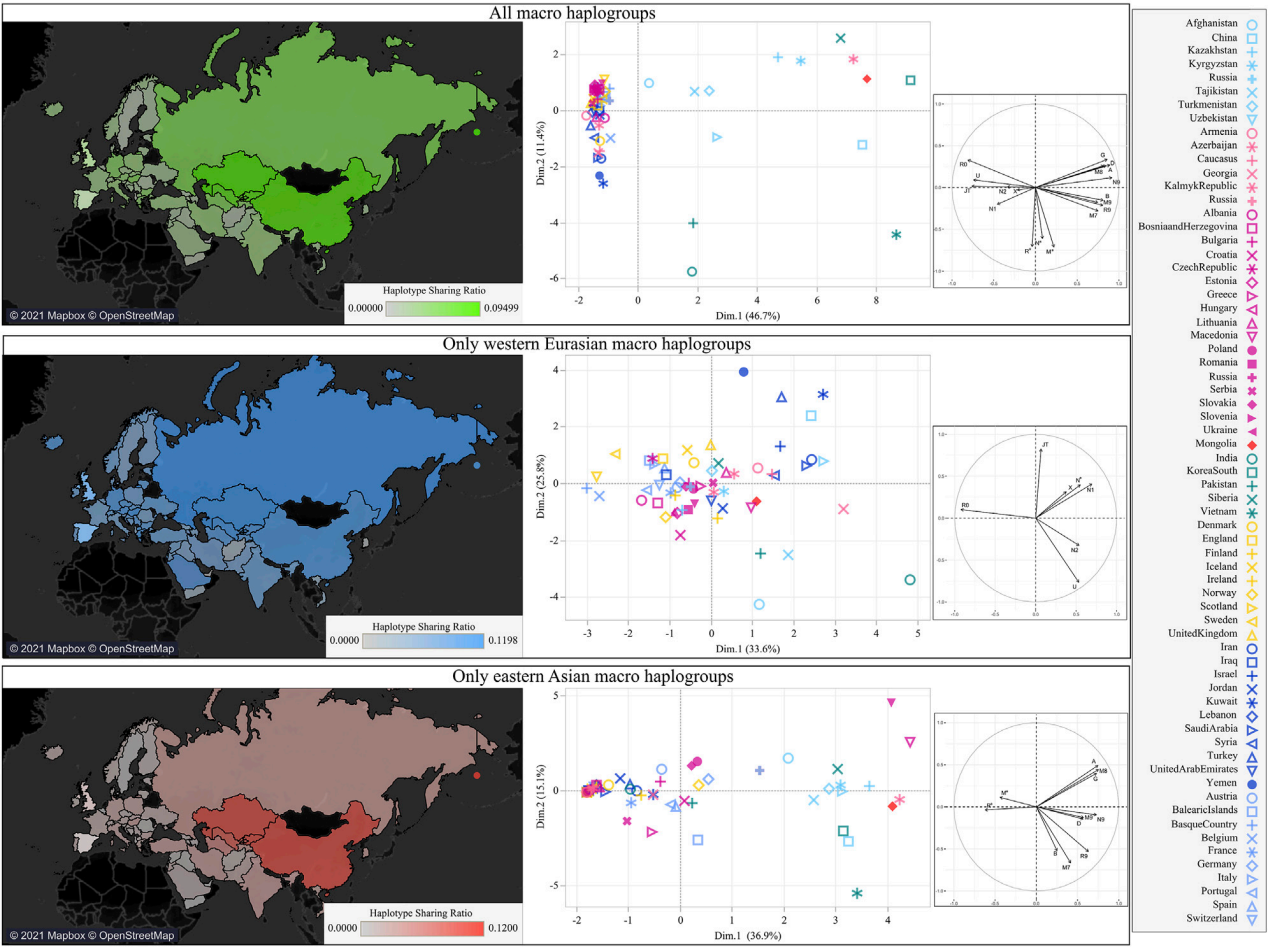
The mtDNA variation of Mongolia in the Eurasian context. Heatmaps based on haplotype sharing between Mongolia and other Eurasian populations are shown on the left. PCA plots based on macro-haplogroup frequencies are shown on the right. All lineages were included in the top panel, the differential contributions of WEu and EAs lineages were explored in the middle and lower panel, respectively.

Considering the various mtDNA contributions to different regions within the country, we evaluated the Mongolian mitochondrial gene pool in the Eurasian context through a MDS plot based on genetic distances, detecting an outlier behavior (Supplementary Figure S1). This peculiarity was further investigated through the analysis of haplotype sharing and by building a Eurasian PCA based on macro-haplogroup frequencies (Figure 2). The map based on haplotype sharing shows a greater proximity to surrounding Asian populations. When we tried to differentiate eastern and western contributions, the legacy of WEu haplogroups was less marked and more widespread. Therefore, it is apparent that the genetic relationships with neighboring populations were mostly driven by EAs lineages and the higher haplotype sharing suggests continuous interactions.

### The Mitochondrial DNA Variation in Mongolia Based on Complete Mitogenomes

To deepen the understanding of mtDNA peculiarities of Mongolians, we extended the analysis to the maximum level of resolution. A total of 147 complete mitogenomes were obtained, including 26 mtDNAs representative of EAs lineages (B, C, D, F, M*, R1, R2, R11) and 121 belonging to WEu haplogroups (H, HV, I, J, K, T, U, W, X) (Supplementary Figure S2; Supplementary Table S4). Through the phylogenetic analysis of our complete mitogenomes, we identified two novel mtDNA sub-branches of haplogroups HV and U5. Three different HV haplotypes from the provinces of Dornod, Khövsgöl and Uvs showed common mutational motifs both in coding (at nps 1654, 9377 and 11152) and control regions (at nps 16184 and 16291) in addition to HV13b motif, thus allowing us to classify these sequences into a postulated haplogroup HV13b1. Four Mongolian individuals from Khentii, Khovd and Zavkhan presenting three different haplotypes were classified as U5b1c2, due to a common transition at np 9110. The entire haplogroup H1 was represented in our control-region dataset with a frequency three times higher (∼3%) than the value reported in literature for Inner Asia (Ottoni et al., 2010) and through complete mitogenome analysis, we identified different sub-clades: H1b and H1c, previously found in Asia as well as in Europe, and H1j, that is very uncommon in the Asian continent.

**FIGURE 3:**
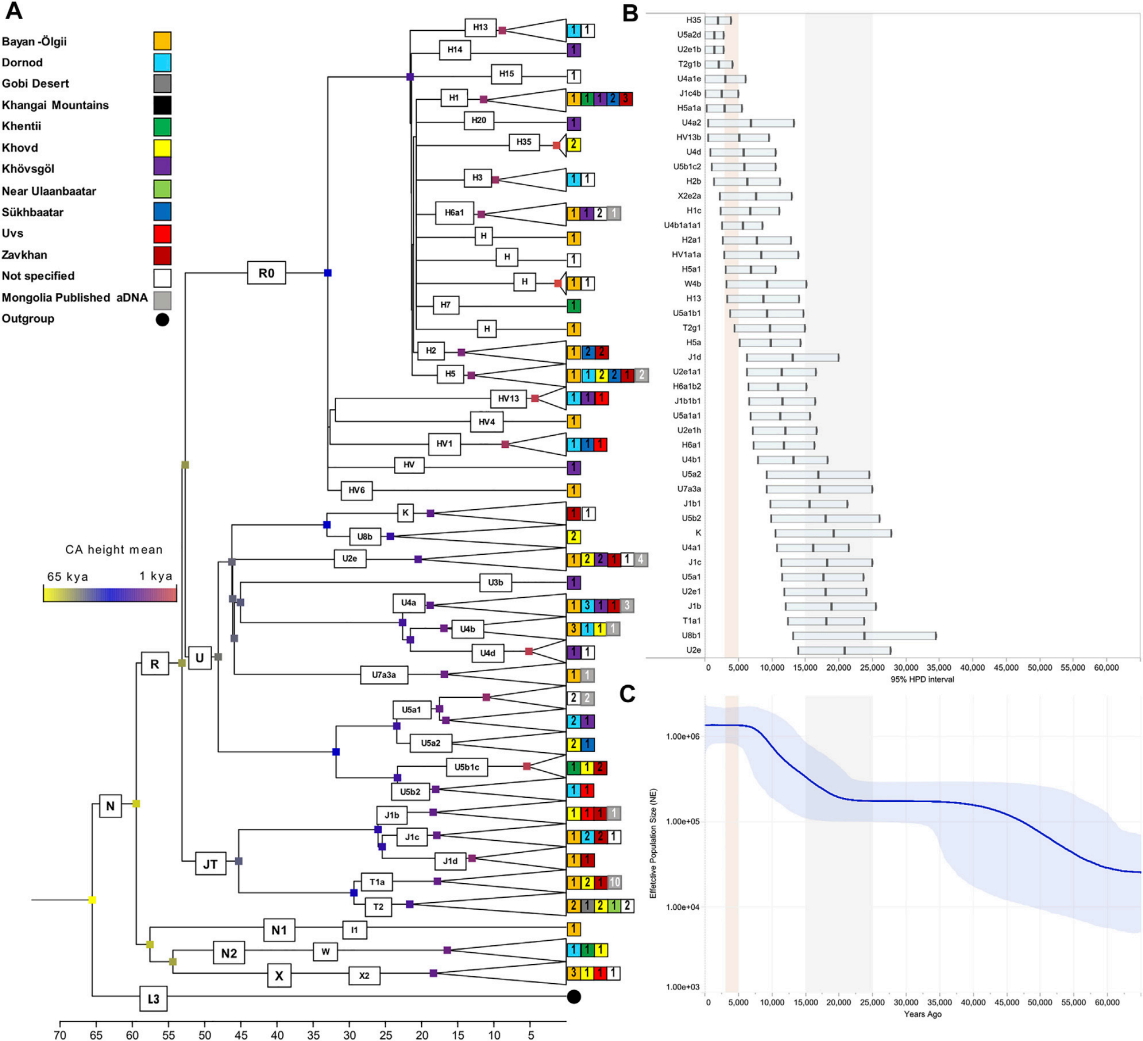
The mtDNA variation in Mongolia based on 121 (new) modern and 25 (published) ancient complete mitogenomes belonging to WEu haplogroups. **(A)** Bayesian tree with internal nodes colored according to common ancestor (CA) average age estimates. **(B)** Age estimates and 95% high posterior densities. LGM timeframe is highlighted in grey, while the red shade indicates Bronze Age. **(C)** Bayesian Skyline Plot (BSP) displaying changes in the effective population size (Ne) through time and considering a generation time of 25 years.

**FIGURE 4:**
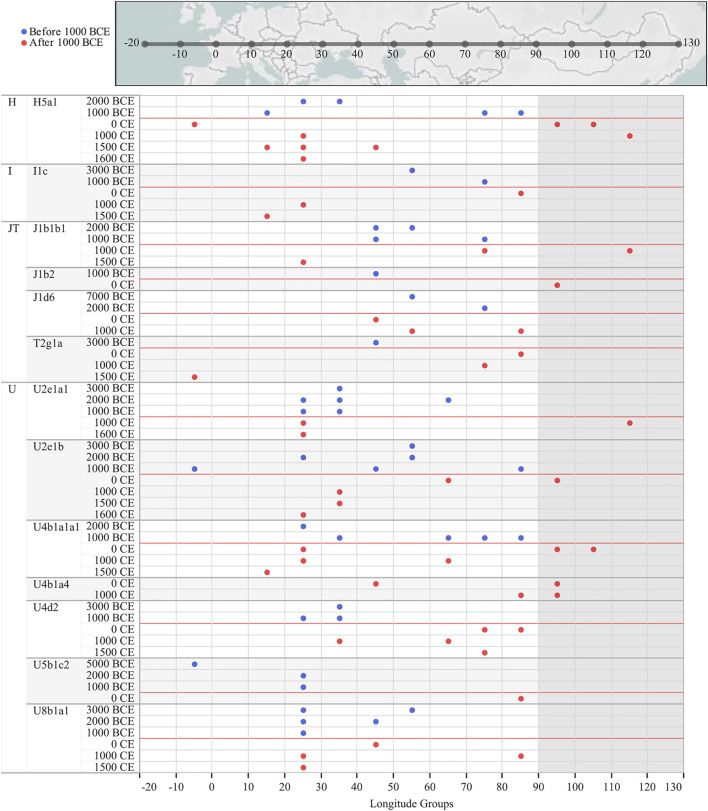
Ancient mitogenomes typical of western Eurasia that were identified among contemporary Mongolians and in ancient remains excavated in Mongolia as well as in other Eurasian regions to the west. A longitude axis is indicated at the bottom and in the geographic map on the top. Mongolia longitudes are shaded. A timeline of 1000 years BCE is reported in red and only those lineages identified in ancient individuals from Mongolia (or nearby regions) and dated after this timeline are reported; see Supplementary Figure S3 and Supplementary Table S5 for the entire dataset.

In order to provide a timeframe to the mtDNA inputs from the west, we focused on the WEu mitogenomes providing Bayesian coalescence ages of internal nodes. Most of these lineages, with a western Eurasian origin but now also identified in Mongolia, coalesced during and soon after the last glacial maximum (LGM, ∼25–15 kya; purple nodes) (Figures 3A,B). The BSP describes a demographic trend with two major increases of the effective population size (Ne; Figure 3C). The first one between 60 and 45 kya likely reflects the initial increase of Ne due to the initial settlement of Eurasia by modern humans during the Pleistocene after the Out-of-Africa exit. Another major increment of Ne seems to be characterized by two steps: the first one started in the late glacial period ∼18–15 kya, while the second took place in early Holocene and was probably facilitated by ecological changes associated with the Holocene Climatic Optimum (HCO; ∼10–6 kya) (An et al., 2008; Orkhonselenge et al., 2018). When considering the 95% HPD, we noticed that some lineages started to coalesce in the early Bronze Age (∼5 kya). They were probably carried by nomadic steppe populations but did not contribute significantly to the Ne. Finally, a very few haplogroups originated in more recent times (<3 kya) and could be linked to historical events. The latter timeframe cannot be accurately described through coalescent estimates taking into account that mitogenomes accumulate one mutation in more than two thousand years on average (Soares et al., 2009; Posth et al., 2016). Therefore, we tried to temporally and geographically reconstruct possible routes marked by the WEu lineages of modern Mongolians, by building a map with the ancient mitogenomes belonging to these lineages that were identified in ancient remains excavated in Mongolia (and nearby regions) as well as in other Eurasian regions to the west. Considering a post-Bronze Age timeline of one thousand years BCE, it is clear that the majority of these WEu lineages were already present in Mongolia in prehistoric times (Supplementary Figure S3). However, other WEu lineages (H5a1, I1c, J1b1b1, J1b2, J1d6, T2g1a, U2e1a1, U2e1b, U4b1a1a1, U4b1a4, U4d2, U5b1c2, U8b1a1) reached Mongolia (or nearby regions) after 1000 BCE (Figure 4) after appearing in areas 20–40 degrees of longitude to the west.

## Discussion

Mongolia is one of the most sparsely populated countries in the world, but complex population interactions occurred across this Eastern Steppe region over the past several millennia (Schurr and Pipes, 2011). Here we characterized the mtDNA of 2,420 modern individuals with a TMA from Mongolia. Our analyses showed a high mitochondrial variation that is heterogeneously distributed across the country. The higher diversity values are present in the northern regions with a dramatic increase in the cosmopolitan area near the capital Ulaanbaatar. If the first finding agrees with reported paleogenomic data (Jeong et al., 2019), the peculiarity of the area around the capital could be better explained by more recent migrations that probably wiped out the original mtDNA gene pool. Other populations probably remained more isolated due to geographic barriers (mountains and desert), which reduced the number of different mitogenomes since ancient times (e.g., in the Khangai Mountains) or only recently (e.g., in the Khovd region).

The majority of mtDNAs belong to haplogroups typical of eastern Asian populations whose frequency decreases from eastern to western regions. An opposite pattern could be observed for those lineages characteristic of western Eurasia. Overall, both EAs and WEu haplogroups contributed to create the mtDNA differentiation currently detectable in Mongolia, as highlighted by the PCA (Figure 1). The WEu lineages determine the genetic distinction of the three westernmost provinces (Bayan-Ölgii, Khovd and Uvs), mostly due to macro-haplogroups JT, R0 and X. In particular, macro-haplogroup R0 (mostly made of H mtDNAs, 36.1%) characterizes the outlier position of people living in the Bayan-Ölgii province, which encompasses the Altai Mountains. The Altai Mountains have initially been considered a genetic barrier to gene flows from the west until the recent discovery of ancient people with a WEu mtDNA living on the Mongol Steppe east of the Altai Mountains before the Bronze Age (Rogers et al., 2020). Different EAs lineages distinguish the southern regions, while the northeastern provinces (Dornod, Khentii, Khövsgöl and Sükhbaatar) cluster together, separately from the others, and are characterized by a high number of different mitogenomes that arrived mostly from the surrounding eastern Asian countries. Actually, genetic closeness and continuous interactions of Mongolia with neighboring populations are witnessed by the shared haplotypes of typical EAs lineages. During Early and Late Medieval time, these interactions across the east Asian Steppe were probably facilitated by a series of organized and highly influential nomadic empires, which had a major impact on the demography and geopolitics of Eurasia until the fall of the Mongol Empire (Jeong et al., 2020). A different pattern, more homogeneous and widespread, has been observed when considering shared haplotypes belonging to WEu lineages without pointing to any apparent connection. Therefore, we pushed the analysis to the highest level of resolution, by considering the information hidden in complete mitogenomes and reconstructing and dating the phylogenetic tree of western Eurasian haplogroups found in Mongolia. The ages of some lineages fall in late and post-glacial times and the demographic analysis highlighted a significant increase of mtDNA lineages and population size that started right after the LGM and became steeper during the HCO, probably marking two different demographic events. The first reflects post-glacial re-populations from glacial refuges in western Eurasia, as testified by the haplotype sharing with contemporary populations from Europe and the Balkans peninsula (a well-known refuge area during LGM), and the high frequency of haplogroup H1, which was indicated as a genetic marker of the post-glacial expansions from western European refuge areas (Achilli et al., 2004). A post-glacial expansion in eastern Asia was already proved for another mtDNA post-glacial marker, haplogroup U5b (Achilli et al., 2005). A later expansion can be probably connected to the climatic amelioration of the early Holocene that was accompanied by the development of farming and pastoralism and more sedentary communities. A mixed ancestry between Yamnaya and European farmers was recently identified by analyzing ancient Bronze Age Mongolians (Jeong et al., 2020; Wang C. C. et al., 2021). We could not identify sub-branches of WEu lineages specific to Mongolia. Therefore, most of the WEu lineages detected in modern Mongolians actually evolved in western Eurasia, and the increments of the population size depicted by our BSP might mirror demographic events that took place in regions to the west of Mongolia. The lack of Mongolia-specific sub-branches might also suggest that the WEu lineages arrived in the Eastern Steppe in more recent times. Certainly, the ages of some WEu lineages between 5 and 3 kya could be linked to Bronze Age migrations across the Eurasian steppes that probably involved also the Afanasievo first (ca. 3300–2500 BCE) and later the Sintashta culture (ca. 2100–1800 BCE). Finally, by searching the available database of ancient mitogenomes for WEu lineages identified in our modern Mongolians, we identified 13 different sub-lineages among remains excavated in Mongolia and dated after the Bronze Age. They might testify for small population movements from the west less than 3,000 ya that can be probably related to commercial routes. Actually, the migration path from western Eurasia to Mongolia marked by some of these mitochondrial sub-lineages (H5a1, J1b2, T2g, U2e1b, U4b1a1a1, and U4b1a4) occurred about 2,500 ya, thus temporally and geographically overlapping with the Silk Route, while other sub-haplogroups, such as J1b1b1 and U2e1a1, seem to have arrived in Mongolia later.

## Conclusion

The gene pool of present-day Mongolians reflects gene flows and demographic processes that occurred over the past several millennia across the Eurasian Steppe, thus representing an important key to reconstruct the genetic history of Inner Asia as well as western Eurasia. The analyses of a large set (N = 2,420) of mtDNAs allowed us to identify peculiarities of the mitochondrial gene pools of different Mongolian regions. A clear matrilineal differentiation was identified across the country due to the differential contribution of mitochondrial lineages of eastern Asian and western Eurasian origins. The EAs contribution was probably linked to continuous interactions with neighboring regions until present days, presumably including those related to the Mongol Empire expansions (1206–1368 CE). The inputs from the west were more difficult to pinpoint. Therefore, we moved to the analysis of entire mitogenomes (N = 147), which allowed us to date the WEu lineages and to reconstruct demographic trends across time. After the first migration of Paleolithic hunter-gatherers, the major increases of the population size could be linked to post-glacial late Pleistocene expansions and to changes towards a more sedentary lifestyle during the Holocene. However, the lack of Mongolia-specific lineages did not allow to directly study mitogenomes that evolved *in situ*. A few and more recent events have been also reconstructed through the analysis of modern and ancient mitogenomes, some during the Bronze Age, others in the last three thousand years. As if haplogroup H1 (and its sub-clades) might suggest a direct link between Europe and Mongolia, six sub-lineages identified in ancient mitogenomes perfectly match the timeframe and path of the Silk Route and can be still identified in present-day Mongolians. Finally, rather than finding long-distance traces of the Mongol Empire expansion to the west, we identified continuous and recent (female-mediated) connections with neighboring Eastern Asian populations. The geographically restricted sharing of haplotypes from typical EAs mtDNA lineages might represent an outcome of Genghis Khan’s so-called *Pax Mongolica* still detectable in present-day Mongolians.

## Data Availability

The original contributions presented in the study are publicly available. This data can be found here: OL632312–OL634731 for mtDNA control regions and OL619795–OL619941 for complete mitogenomes.
