# Supplementary material for: Mitochondrial DNA Footprints from Western Eurasia in Modern Mongolia
Source: Front Genet. 2022 Jan 6;12:819337. doi: 10.3389/fgene.2021.819337 (PMC8773455; doi:10.3389/fgene.2021.819337)
Supplement: Supplementary file 1 [file DataSheet1.PDF]

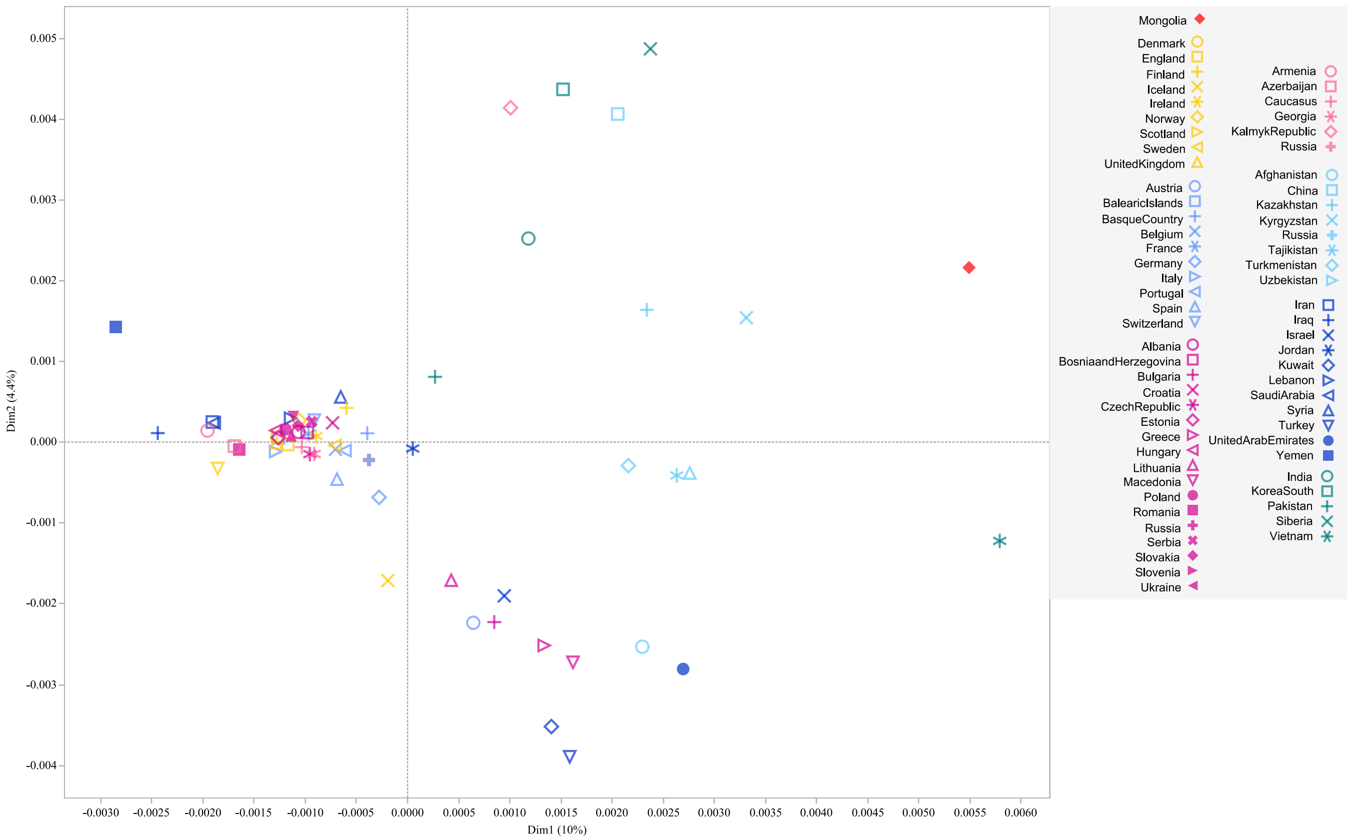

Figure S1. Multidimensional Scaling (MDS) plot generated including our Mongolian control-region data and all the available Eurasian mtDNAs (N=30400; see Supplementary Table S3).

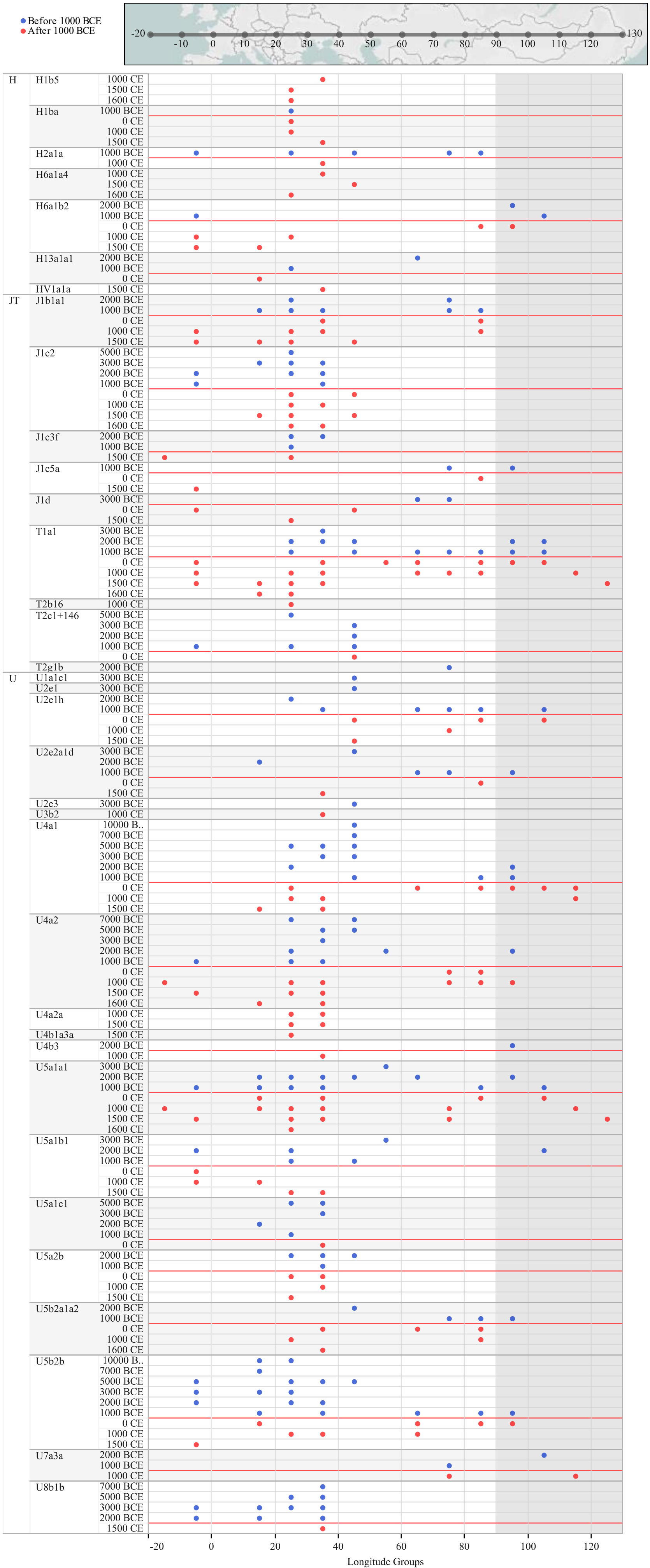

Figure S3. Ancient mitogenomes typical of western Eurasia that were identified among contemporary Mongolians and in ancient remains excavated in Mongolia as well as in other Eurasian regions to the west. A longitude axis is indicated at the bottom and in the geographic map on the top. Mongolia longitudes are shaded. A timeline of 1000 years BCE is reported in red; see also Figure 3 and supplementary Table S5 for the entire dataset.
